# Virtual reality distraction induces hypoalgesia in patients with chronic low back pain: a randomized controlled trial

**DOI:** 10.1186/s12984-020-00688-0

**Published:** 2020-04-22

**Authors:** Thomas Matheve, Katleen Bogaerts, Annick Timmermans

**Affiliations:** 1 Faculty of Rehabilitation Sciences, Hasselt University, Agoralaan, building A, 3590 Diepenbeek, Belgium; 2 Health Psychology, University of Leuven, Leuven, Belgium

**Keywords:** Distraction, Chronic low back pain, Virtual reality, Analgesia, Pain-related fear, Catastrophizing, Gamification

## Abstract

**Background:**

Attentional distraction from pain has been shown to be largely ineffective for obtaining a hypoalgesic effect in patients with chronic pain when compared to a control condition. It has been hypothesized that this may be due to the non-engaging types of distraction that have been used so far. Moreover, it is suggested that the hypoalgesic effects of distraction may be attenuated by pain-related cognitions and emotions, as they may increase the attention to pain.

**Methods:**

In this randomized controlled trial, patients with chronic nonspecific low back pain in the intervention group (*n* = 42) performed a single exercise session with nonimmersive VR games, while those in the control group (*n* = 42) performed the same exercises without VR games. We investigated whether VR distraction had a hypoalgesic effect during and immediately after the exercises, and whether it reduced the time spent thinking of pain during the exercises. We further explored whether pain-related fear, pain catastrophizing and baseline pain intensity moderated the effects of VR distraction.

**Results:**

VR distraction had a hypoalgesic effect during (Cohen’s d = 1.29) and immediately after (Cohen’s d = 0.85) the exercises, and it also reduced the time spent thinking of pain (Cohen’s d = 1.31). Preliminary exploratory analyses showed that pain-related fear, pain catastrophizing and baseline pain intensity did not moderate the effects of VR distraction.

**Conclusions:**

Large effect sizes of VR distraction induced hypoalgesia were observed. This suggests that nonimmersive VR games can be used when it is deemed important to reduce the pain during exercises in patients with chronic nonspecific low back pain.

**Trial registration:**

NCT02679300. This trial was registered on 10 February 2016.

## Background

Attentional distraction, defined as shifting the attention away from the pain, is a commonly used strategy in pain management [1, 2]. The hypoalgesic effects of distraction have mainly been investigated during experimentally induced pain in healthy persons or during acute procedural pain (e.g. needle pain). Although some of these studies have failed to demonstrate a hypoalgesic effect of distraction [3, 4], the majority of studies have shown that distraction does reduce pain in these populations [5–9]. However, a recent meta-analysis reported that distraction did not have a hypoalgesic effect in patients with chronic pain when compared to a control condition without specific instructions [2].

Various hypotheses have been postulated to explain the lack of distraction induced hypoalgesia in patients with chronic pain. For example, patients with chronic pain have been shown to selectively pay attention to pain-related information [10, 11], and as a consequence, they might be less easily distracted from it [2, 12]. This attentional bias to pain develops when the pain is experienced as threatening [10], and as such, this bias is often associated with higher levels of pain-related fear and pain catastrophizing [10, 11]. However, the moderating effects of pain-related cognitions and emotions on the effectiveness of distraction in patients with chronic pain are equivocal [13–17]. Further, it is hypothesized that the type of distraction may play a role. Evidence is emerging that the attention to pain should be considered from a motivational perspective [5, 18]. It is thought that in order to draw the attention away from the pain, the competing stimulus should be sufficiently engaging [19]. In this respect, interactive virtual reality (VR) games may prove to be a promising tool [20–24], as they are typically considered to be motivating [25].

In some randomized controlled trials, VR games have already been integrated into rehabilitation programs for patients with chronic pain [26–28]. However, none of these studies have specifically investigated whether patients experienced less pain while being immersed in a VR environment. As such, the available evidence showing that VR distraction may have a hypoalgesic effect in patients with chronic pain almost exclusively comes from uncontrolled studies with small sample sizes [29–38], so firm conclusions cannot be drawn. In addition, the potential influence of pain-related cognitions and emotions on the hypoalgesic effects that VR distraction may have on chronic pain has not been investigated [39]. Therefore, more research is needed to address these shortcomings.

In this study, patients with chronic low back pain (CLBP) performed exercises during a single intervention session. *First*, we investigated whether performing these exercises in a nonimmersive VR environment had an influence on the pain intensity and the time spent thinking of pain during the exercises, when compared to a control group who performed the same exercises without VR distraction. *Second*, the pain intensity immediately after the exercises was assessed because distracting patients with CLBP during an active task may paradoxically increase the pain intensity after this task [15]. *Finally*, for exploratory purposes we assessed the influence of baseline measures of pain intensity, pain-related fear and pain catastrophizing on the effectiveness of the VR distraction.

## Methods

### Participants

Eighty-four patients who were undergoing active treatment were included. Inclusion criteria were an age of 18 to 65 years old, sufficient knowledge of the Dutch language, a diagnosis of chronic non-specific low back pain (> 3 months, ≥3 days/week) by a physician, a baseline pain score between 3 and 8 on a 0 to 10 numeric pain rating scale and the ability to perform pelvic tilt exercises in a standing position. Participants were excluded if they had previous spinal surgery, recent (< 6 months) spinal infiltrations, signs or symptoms of nerve root involvement, an underlying serious pathology (e.g. multiple sclerosis), fibromyalgia, confirmed or suspected pregnancy and experience with VR rehabilitation. All participants gave written informed consent before being included in the study. Ethical approval was obtained from the Ethics Committees of Hasselt University and Jessa Hospital, Belgium (approval number 15.128/REVA15.11).

### Recruitment and randomization

Recruitment was performed at the Jessa Hospital (Belgium) and took place between February 2016 and November 2018. At the moment of recruitment, all subjects were participating in an outpatient rehabilitation program for CLBP. After baseline assessment, participants were randomized in a VR-group or a control group. For this randomization, a research assistant that was not further involved in this study used a computer-generated sequencing system that produced a list of numbers between 1 and 84 in random order (each number only appeared once in the list). Even numbers corresponded with the VR-group, whereas uneven numbers corresponded with the control group. Based on this random sequence, the research assistant prepared sequentially numbered, sealed opaque envelopes containing a piece of paper that indicated to which group a person would be assigned. After baseline assessment, the researcher that conducted the experiments opened the first envelope to see to which group the participant would be assigned.

### Intervention

We used a single-session intervention, which consisted of 2 × 2 min of pelvic tilt exercises in the sagittal plane, with 30 s of rest in between. Pelvic tilt exercises are often used in the rehabilitation of patients with CLBP, and their purpose is to (re)gain movement control of the lumbar spine and pelvis [40, 41]. The latter can be important, as it has been suggested that poor movement control may be an underlying mechanism contributing to the persistence of CLBP [42]. In the current study, the pelvic tilt exercises were performed in a standing position with slightly bent knees and participants placed their hands on the side of their pelvis to guide the pelvic movements.

Upon arrival, participants were explained that they first had to complete various questionnaires, after which they would be asked to perform pelvic tilt exercises for a few minutes. Participants were told that after the exercises, they would have to answer a few short questions concerning their experiences with the exercises. No reference was made to our interest in pain intensity or the potential effect of VR distraction. Just before the start of the intervention, participants received instructions on how to perform the pelvic tilts. The experiments took place in a separate room in the hospital, with no other patients present. During the experiments, an investigator was present in the room and sat a few meters behind the participant, outside the participant’s field of vision. During the intervention, the investigator visually checked whether the exercises were performed correctly, but no feedback was provided. If participants failed to perform the exercises in a correct way (e.g. by making whole-body movements instead of pelvic tilts), they would be excluded from further analyses. As such, both the participants and the investigators were not blinded to the intervention.

#### Virtual reality group

**Fig. 1:**
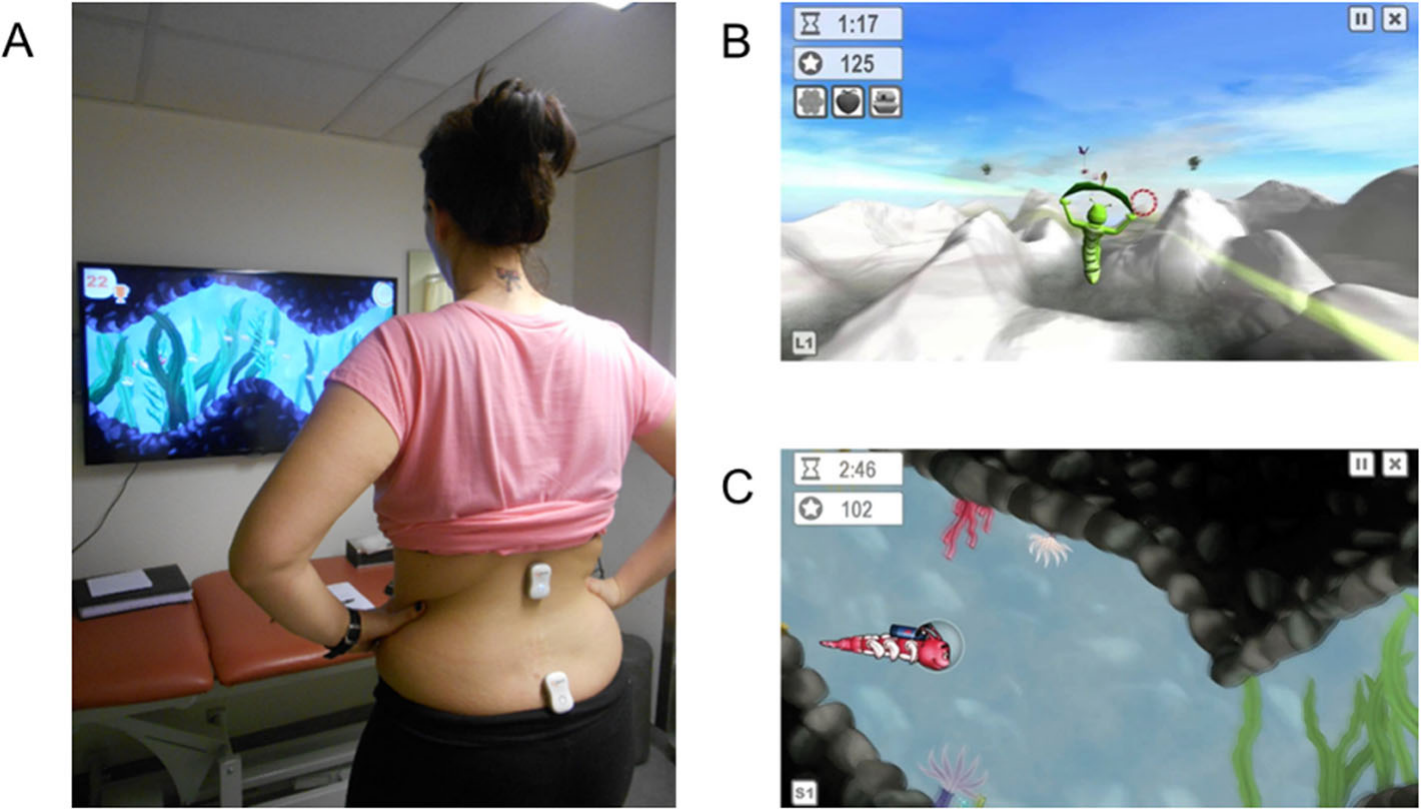
**a**: Participant playing the VR games. **b**-**c**: VR games

The VR group played 2 different games (2 min each), which had to be controlled by pelvic tilts in the sagittal plane. To play the games, a wireless motion sensor (Valedo®Pro, Hocoma, Switzerland) was placed on the sacrum at the S2-level using double sided tape (an additional sensor at the L1-level was used for calibration of the system). The sensor measures with an accuracy of 0.1° and a frequency of 50 Hz. The sensor-signals were sent to a laptop that was connected to a high definition TV screen (47 in.) on which the games were displayed. During the games, participants stood in front of TV-screen at a distance of 2.5 m and the sound of the TV was turned on (Fig. 1a). The goal of the first game was to collect points by guiding a caterpillar through hoops and by catching objects that were floating in the air (Fig. 1b). During the second game, points could be gained by guiding a fish through a cave and to collect shells, while avoiding hitting the cave walls or bumping into other fish (Fig. 1c). The caterpillar and fish could be steered upwards or downwards by tilting the pelvis in an anterior or posterior direction. Before the start of the intervention, the purpose of the games was explained and participants were instructed how to play them (i.e. with pelvic tilts).

#### Control group

The control intervention was conducted in the same room as the VR intervention. During the control intervention, participants stood in exactly the same spot as those in the VR group, but with the TV screen switched off. The control group performed pelvic tilts in the sagittal plane according to a beep tone. The first time participants heard the tone, they had to their pelvis anteriorly and keep it in an anteriorly tilted position until the next beep, after which participants tilted the pelvis posteriorly, and so on. Participants had to tilt their pelvis 46 times during the first two minutes, and 54 times during the second two minutes. The tempo of the beep tones was varied, so that participants sometimes had to tilt their pelvis faster or slower. The number and the tempo of the beep tones were based on a pilot trial that was conducted prior to the current study. During this pilot trial, 12 persons fulfilling the criteria of the current study performed the VR-games as described above, while the movements of the pelvis were recorded with a digital video camera. Two research assistants, not further involved in the study, counted the number of pelvic tilts and assessed the tempo of the pelvic movements independently from each other. Based on these observations, audio files for each of the two games were created prior to the main study in order to mimic the number and tempo of pelvic tilts during the VR-games as closely as possible. The participants of the pilot trial were not included in this study.

### Assessments and outcome measures

#### Baseline assessments

##### Sociodemographic data

Participants were asked to provide information on their age, weight, height, sex and duration of LBP.

##### Numeric pain rating scale (NPRS) [43]

Participants were asked to indicate the average intensity of their LBP over the past 7 days and the intensity of their current LBP on a 0 to 10 numeric rating scale (0 = no pain, 10 = worst imaginable pain). The 11-point NPRS has been widely used to measure pain intensity in a CLBP population and has been recommended to be used in clinical trials [44, 45].

##### Roland Morris disability questionnaire (RMDQ) [46]

The RMDQ contains 24 questions about the effect of LBP on daily activities, which have to be answered with yes or no. A higher score (range 0–24) represents a higher level of disability. The RMDQ has been shown to be valid and reliable in a CLBP population [43].

##### Pain Catastrophizing scale (PCS) [47]

The PCS contains 13 statements relating to the patients’ negative thoughts and feelings during pain. Each statement has to be answered on a 5-point scale (0 = not at all, 4 = always), resulting in a score between 0 and 52. A higher score corresponds with a higher level of pain catastrophizing. The PCS is valid and reliable in a CLBP population [48]. Cronbach’s alpha of the PCS in this study was 0.91.

##### Tampa scale for Kinesiophobia (TSK) [49]

The TSK is a questionnaire containing 17 items to assess subjective ratings of fear of movement/re-injury due to physical activity. The total score ranges between 17 and 68, with a higher score indicating a higher level of kinesiophobia. The TSK is valid and reliable in patients with CLBP [43]. Cronbach’s alpha of the TSK in this study was 0.75.

#### Post-intervention assessments

Immediately after the exercises, participants completed the following three questions on a 0 to 10 numeric rating scale. Question one: ‘What was the average intensity of your low back pain during the exercises?’ (0 = no pain, 10 = worst imaginable pain). Question two: ‘How much did you think of your low back pain during the exercises?’ (0 = not at all, 10 = all the time) [50]. Question three: ‘What is the intensity of your low back pain at this moment?’ (0 = no pain, 10 = worst imaginable pain). These assessments were performed after the intervention in order not to create conflicting attentional processes, i.e. asking participants to report pain during the exercises while the purpose of the VR-intervention was to distract them from the pain [2].

After the abovementioned questions, two additional questions were asked. Based on previous research [25], the VR games used in this study were expected to be motivating. To confirm this, participants in the VR-group were asked to answer the following question, which was derived from the Immersive Experience Questionnaire [51]: ‘To what extent did you feel motivated while playing the virtual reality games?’ (1 = not at all, 7 = a lot). Second, we assumed that participants would not perceive the exercises as harmful. We checked this assumption, because the perceived harmfulness during a movement task has been shown to be related to the pain experience during that task [52]. Therefore, participants in both groups were asked to answer the following question: ‘How harmful did you think the exercises were for your lower back?’ (0 = not harmful at all, 10 = extremely harmful).

#### Outcome measures

##### Primary outcome

The difference between baseline pain intensity and the pain intensity experienced during the exercises was the primary outcome. This difference was obtained by subtracting the pain intensity during the exercises from the baseline pain intensity. A positive value thus indicates an improvement.

##### Secondary outcomes

(1) The difference between baseline pain intensity and the pain intensity experienced immediately after the intervention, (2) the time spent thinking of pain during the exercises and (3) the number of pelvic tilts performed by the participants were the secondary outcomes. The pain difference after the exercises was obtained by subtracting the pain intensity after the exercises from the baseline pain intensity. A positive value indicates an improvement. The number of pelvic tilts in both groups was assessed in the same way as during the pilot trial. If between group differences would be present, or the number of pelvic tilts would be correlated with any of the outcomes, this factor would be controlled for in the analyses.

### Sample size calculation

Because this is the first randomized controlled trial investigating the hypoalgesic effects of VR distraction in patients with CLBP, and similar studies in other chronic pain populations are lacking, we based our sample size calculation on effect sizes reported in meta-analyses including studies on experimentally induced and acute procedural pain. Large effect sizes of VR distraction for pain reduction ranging between 0.9 [23] and 0.94 [53] were reported in these meta-analyses. However, when only the effects on clinical pain were considered, Kenney et al. [23] reported a smaller effect size of 0.62. Given that we assessed clinical pain in the current study, the most conservative estimate of 0.62 for the primary outcome measure (pain improvement during the distraction) was used. Together with an alpha-level of 0.05 and power of 0.8, a total of 84 participants needed to be included in this study.

### Statistical analyses

All statistical analyses were performed with JMP Pro version 14.1 (SAS institute, Cary, NC). Data fulfilled all the assumptions for performing parametric statistical analyses. To investigate the effects of VR distraction on the pain intensity, a repeated measures ANOVA was performed with group (VR vs control) as between groups factor and time (difference in pain during exercises vs difference in pain after exercises) as within group factor. The effects of VR distraction on the time spent thinking of the pain were assessed with a t-test for independent samples.

The influence of pain-related fear, pain catastrophizing and baseline pain intensity on the effectiveness of VR distraction was assessed using both continuous and dichotomized scores on these questionnaires. Using dichotomized scores lowers the power to detect significant effects, but it facilitates the clinical interpretation of the results [54]. Therefore, we used both approaches to analyze our data. The dichotomization of the baseline measures into ‘low’ and ‘high’ groups was done according to Linton et al. [55] First, we performed a median split of the TSK, PCS and pain intensity scores, after which we compared them to previously reported clinically relevant cut-off values [55]. For both groups in the current study, the median scores for the TSK and PCS were 37 and 22, respectively. The clinically relevant cut-off values reported in the literature for the TSK range between 37 and 40, whereas for the PCS these scores range between 22 and 30 [47, 55–58]. Given that the median scores of the current study fell within these ranges, albeit on the lower end of the spectrum, we continued using the median splits. The median baseline pain score in the current study was 4.5/10 for both the VR and control group. Pain scores are often separated into three categories, and in patients with LBP, a score on the NPRS between 1 and 4 has been reported as mild, between 5 and 6 as moderate and ≥ 7 as severe [59, 60]. Using the median score (4.5/10) of the current study, participants in the low baseline pain groups had scores of 3–4/10 which can be considered as mild pain, whereas participants in the high baseline pain groups had scores between 5 and 8/10, being moderate to severe pain. In the control group, these median splits resulted in mean scores in the respective low and high groups for the TSK of 31.0 (SD = 4.2) and 42.5 (SD = 3.5), for the PCS of 14.9 (SD = 5.6) and 30.3 (SD = 5.3), and for the baseline pain of 3.7 (SD = 0.5) and 6.0 (SD = 0.8). Regarding the VR group, the mean scores in the respective low and high groups for the TSK were 32.9 (SD = 3.0) and 42.3 (SD = 3.2), for the PCS they were 14.5 (SD = 5.9) and 31.6 (SD = 7.7), and for baseline pain intensity they were 3.4 (SD = 0.5) and 6.2 (SD = 0.9). To assess the moderating effects of the baseline measures on the VR distraction, for each dependent variable (pain difference during, pain difference after and the time spent thinking of pain) multiple linear regression models were constructed and 2 × 2 ANOVAs were performed, depending whether the baseline scores were continuous or dichotomous. In all of the regression models and ANOVAs, we included group (VR vs Control) and the baseline measure (i.e. pain-related fear, pain catastrophizing or pain intensity) as main effects, and a group by baseline measure as interaction effect. Post-hoc tests and pre-planned contrast analyses were conducted when appropriate. Partial eta-squared (η_p_^2^) and Cohen’s *d* effect sizes were calculated [61]. The alpha level for statistical significance was set at *p* < 0.05. For the moderation analyses, we used a separate Bonferroni-Holm correction for each baseline measure.

Before running all the analyses, we checked whether the number of pelvic tilts differed between the VR and control group, and whether they were correlated with any of the outcomes in the VR group. Four participants in the control group missed a maximum of 2 pelvic tilts during the intervention, while all of the other participants in the control group performed the set number of pelvic tilts (*n* = 100). The mean number of pelvic tilts performed in the VR group was 98.1 (SD = 15.6), which was not significantly different from the control group (*p* = 0.44). In addition, there was no correlation between the number of pelvic tilts and any of the outcome measures in the VR group (all correlation coefficients ≤0.12, all *p*-values ≥0.44). Therefore, the number of pelvic tilts were not included in further analyses.

## Results

### Participant characteristics

**Fig. 2:**
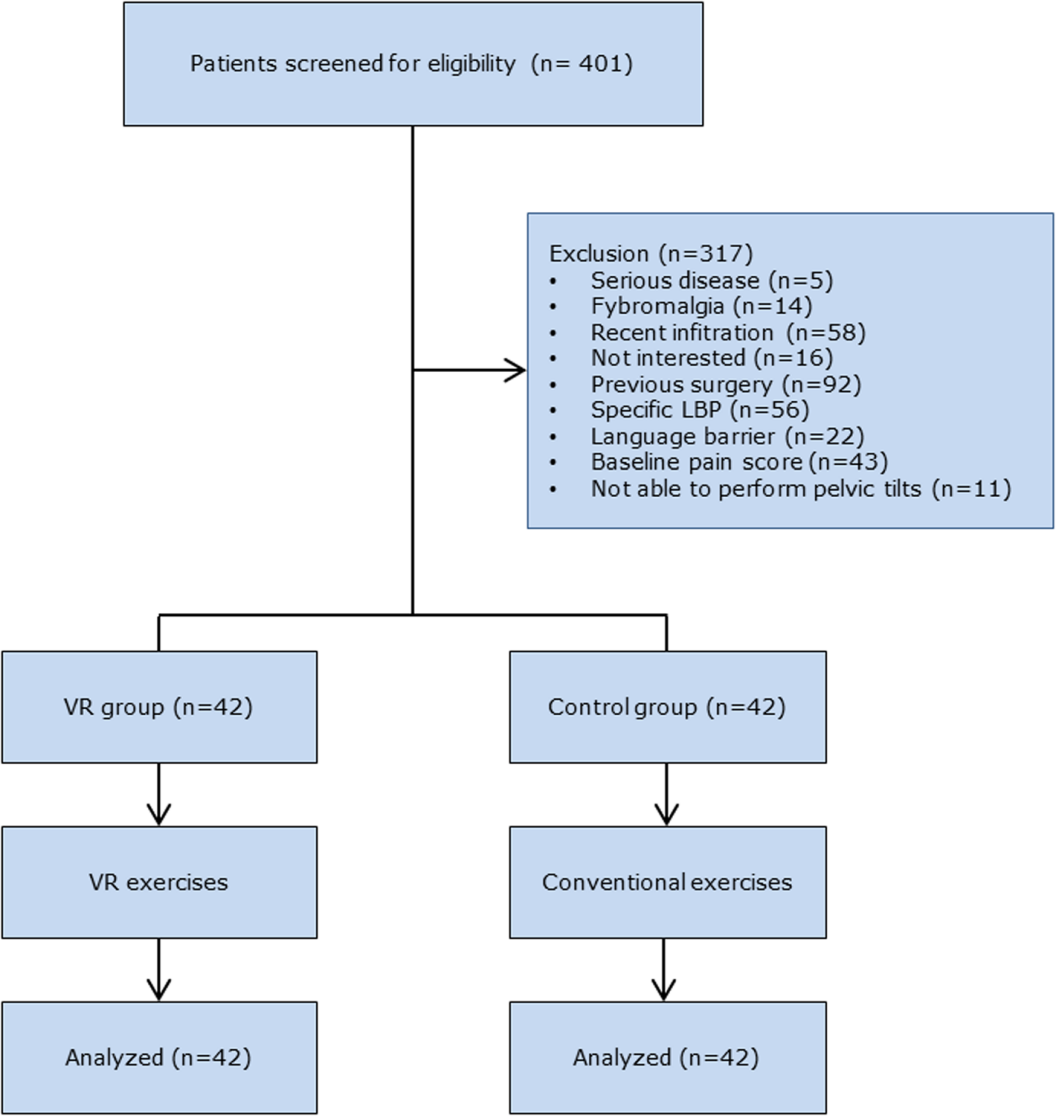
Flowchart of participants

A total of 401 persons were screened for eligibility, of which 84 participants were included. Reasons for exclusion and the flow of participants through the study can be found in Fig. 2. None of the participants had to be excluded because of an incorrect performance of the exercises. An overview of the participants’ characteristics can be found in Table 1. No significant between groups differences were present for any of the baseline measures.

**Table 1:** Baseline characteristics participants

	Control group (*n* = 42)		VR-group (n = 42)		*p*-value
	Mean	(SD)	Mean	(SD)	
Sex *(n female, %)*	27	(64%)	27	(64%)	1.00
Age *(years)*	44.2	(11.9)	42.1	(11.5)	0.41
BMI *(kg/m*^*2*^*)*	26.7	(5.0)	26.8	(4.8)	0.92
Duration LBP *(years)*	10.6	(10.2)	10.8	(10.6)	0.84
Pain past 7 days *(0–10)*	5.5	(1.6)	5.3	(1.6)	0.68
Baseline pain *(0–10)*	4.9	(1.4)	4.8	(1.6)	0.83
RMDQ *(0–24)*	10.9	(4.3)	11.4	(3.8)	0.57
TSK *(17–68)*	36.2	(6.9)	37.0	(5.6)	0.59
PCS *(0–52)*	22.2	(9.4)	22.7	(10.9)	0.86

*BMI* Body mass index, *LBP* Low back pain, *PCS* Pain Catastrophizing Scale, *RMDQ* Roland Morris Disability Questionnaire, *TSK* Tampa Scale for Kinesiophobia

## Effectiveness of VR distraction

### Effects of VR distraction on pain intensity

A repeated measures ANOVA showed a main effect for group (F_(1, 83.6)_ = 28.39, *p* < 0.0001, η_p_^2^ = 0.26) and for time (F_(1, 80.9)_ = 7.42, *p* = 0.008, η_p_^2^ = 0.08), and a group by time interaction (F_(1, 80.9)_ = 9.3, *p* = 0.003, η_p_^2^ = 0.10). Compared to the control group, the VR-group had a significantly larger reduction in pain intensity during the exercises (VR group M = 1.66, SE = 0.25; Control group M = − 0.55, SE = 0.26; Difference = 2.20, SE = 0.36, t_80.9_ = 6.11, *p* < 0.0001, *d* = 1.29 (95% CI = 0.82–1.76)) and after the exercises (VR group M = 0.81, SE = 0.25; Control group M = − 0.50, SE = 0.26; Difference = 1.31, SE = 0.36, t_80.9_ = 3.64, *p* < 0.003, *d* = 0.85 (95% CI = 0.40–1.29)). Raw values for pain intensity and for the differences with baseline can be found in Table 2.

**Table 2:** Raw values for pain intensity and for the differences with baseline

	baseline		During exercises^a^				Post exercises			
	M	SD	M	SD	Difference with baseline		M	SD	Difference with baseline	
					M	95%CI			M	95%CI
Control	4.86	1.37	5.40	1.74	−0.55	− 0.05 to −1.04	5.35	1.74	− 0.50	− 0.04 to − 0.96
VR	4.79	1.61	3.12	2.45	1.67	1.08 to 2.25	3.95	2.41	0.83	0.31 to 1.36

Differences with baseline were calculated as: baseline pain intensity - pain intensity during/post exercises. A positive value thus indicates a decrease in pain intensity during/post exercises relative to baseline. 95%CI = 95% confidence interval, M = mean, SD = standard deviation, VR = virtual reality.

^a^ Primary study outcome: Isolated between-groups comparison (VR vs Control) for the pain difference during, using an unpaired t-test: *p* < 0.0001, t_82_ = 5.83, between groups difference = 2.21 (95% CI = 1.47 to 2.96)

### Effects of VR distraction on the time spent thinking of pain

Participants in the VR group spent significantly less time thinking of their pain during the exercises compared to participants in the control group (VR group M = 2.26, SD = 2.55; Co group M = 5.52, SD = 2.41; Difference = 3.26, t_82_ = 6.01, *p* < 0.0001, *d* = 1.31 (95% CI = 0.84–1.78)).

### Influence of baseline parameters on the effectiveness of VR distraction

Since the results were similar for all three dependent variables (i.e. pain difference during, pain difference after and time spent thinking of pain), we will only provide a summary of results of the ANOVAs in the main text. Further, the factors that were statistically significant in the ANOVAs using the dichotomized scores were identical to those in the regression models when using the continuous scores. Detailed results regarding the results of the ANOVAs, planned contrasts and regression analyses can be found in Additional files 1 and 2, respectively. Because the moderation analyses were exploratory in nature, power calculations for these analyses can be found in Additional file 3.

#### Pain-related fear

Regarding pain-related fear, there was a main effect for group (all *p*-values < 0.0001) and for TSK (all *p*-values < 0.02), but there was no interaction effect (all *p*-values > 0.54) for the outcomes pain difference during exercises, pain difference after the exercises and the time spent thinking of pain. Patients in the low TSK group had larger improvements in pain intensity during and after the exercises, and they thought less of their pain during exercises when compared to the patients in the high TSK group.

#### Pain catastrophizing

Similar results were obtained for pain-catastrophizing. A main effect for group (all *p*-values < 0.0001) and PCS (all p-values < 0.02) was present, but there was no interaction effect (all p-values > 0.58) for all outcomes. Patients in the low PCS group had larger improvements in pain intensity during and after the exercises, and they thought less of their pain during exercises when compared to the patients in the high PCS group.

#### Baseline pain intensity

There was a main effect for group for all outcomes (all p-values ≤0.0002). A main effect for baseline pain intensity was only present for the outcome time spent thinking of pain (*p* < 0.02). No interaction effects were present for any of the outcomes (all *p*-values > 0.75). Patients with lower levels of baseline pain intensity thought less of their pain compared to those with higher levels of baseline pain intensity.

### Motivation and perceived harmfulness

As expected, the participants in the VR group were motivated to play the games. The median motivation score was 6.5/7 (IQR = 6 to 7, minimum score = 5/7). Further, participants in neither group perceived the exercises to be harmful (VR group median score = 0 (IQR = 0 to 2), Control group median score = 0 (IQR = 0 to 3)) and the median harmfulness scores were not significantly different between groups (z = 1.29, *p* = 0.20). Therefore, both of our assumptions regarding the motivation and perceived harmfulness were confirmed.

## Discussion

In this study, we used nonimmersive VR games to distract patients with CLBP during a single exercise session. Our aim was to investigate whether VR distraction had an influence on the pain intensity during and immediately after the exercises, and on the time spent thinking of the pain during the exercises. In addition, the effects of baseline pain intensity, pain-related fear and pain catastrophizing on the effectiveness of VR distraction was assessed. Our results showed that, compared to a control condition, VR distraction significantly reduced the pain intensity during and after the exercises, and also the time spent thinking of the pain. The exploratory moderation analyses showed that, in both the VR and control group, participants with higher levels of pain-related fear and pain catastrophizing experienced a higher pain intensity (or less decrease in pain) during and after the exercises, and spent an increased time thinking of pain. In both groups, a higher baseline pain intensity did not affect the differences in pain intensity during or after the exercises, but it did lead to an increased time spent thinking of pain. Pain-related fear, pain catastrophizing and baseline pain intensity did not moderate the effectiveness of the VR distraction.

The hypoalgesic effect of VR distraction in patients with chronic pain has predominantly been investigated in small and uncontrolled studies [29–34, 37, 38]. Overall, these studies have shown a reduction in pain intensity during and immediately after being immersed in a VR environment. By conducting a randomized controlled trial, we further extended these preliminary findings. A recent meta-analysis showed that distraction did not have a hypoalgesic effect in patients with chronic pain when compared to a control condition [2], which is in contrast to our study. Of importance, none of the studies included in this meta-analysis used VR as a means of distraction. Hence, VR games could potentially be more effective than other types of distraction, which is supported by research on acute procedural pain [20]. One of the suggested reasons for the effectiveness of VR distraction is the motivating character of the games. In the current study, participants were indeed motivated to play the games (minimum motivation score = 5/7). Motivation and goal pursuit have been proposed as important factors affecting the attention to pain. It is suggested that pain will have a less interruptive effect during an ongoing task when a person is motivated to fulfill that task or to achieve a particular goal that is not pain-related [5, 62]. Based on our results, it would be worthwhile to further explore the role of motivation in attentional distraction of patients with chronic pain.

Patients with chronic pain have an attentional bias to pain, although the differences with healthy persons are small and depend on the methods used to measure it [10, 11]. This selective attention to pain and the difficulty to disengage from it can be driven by pain-related cognitions and emotions, such as pain catastrophizing and pain-related fear [63–65]. Therefore, it has been hypothesized that these factors may affect distraction induced hypoalgesia, which is supported by some [2, 17, 66, 67], but not by all studies [15, 16]. However, the preliminary results of our study do not confirm this hypothesis. This may be explained by the fact that we used a motivationally relevant distraction task. Indeed, Verhoeven et al. [5] showed that a neutral non-VR distraction task was effective for reducing experimentally induced pain in low, but not in high catastrophizers. In contrast, the level of pain catastrophizing did not influence the hypoalgesic effects of a motivationally relevant distraction task. Another explanation might be that the exercises in the current study were not perceived as harmful by the participants. Because pain especially captures attention when it is perceived as threatening [12], the effects of VR distraction might be attenuated by pain-related fear or catastrophic thinking if patients have to perform activities they perceive as harmful.

Besides the threat value, also the intensity of pain influences its interruptive effect [12]. This has led to the hypothesis that distraction might be less effective for patients experiencing more severe pain [2]. Few studies have investigated this in a chronic pain population, and the results are equivocal [2, 13]. In our study, baseline pain intensity did not moderate the effectiveness of the VR-distraction. Of importance, the majority of the participants (85%) had mild to moderate levels of baseline pain. As such, only 30% of the patients categorized in the high baseline pain groups had a severe pain intensity at baseline (i.e. NPRS score of 7–8/10), which might have limited our ability to detect a moderating effect of this parameter.

Apart from the abovementioned reasons that may explain the lack of moderation, it should also be kept in mind that, for most of these analyses, our study was not sufficiently powered to detect small effects (i.e. η_p_^2^ < 0.01). As such, these results should be interpreted cautiously. In order to be able to detect small moderation effects, studies with larger samples are necessary.

A significant amount of research on the hypoalgesic effects of (VR) distraction has been done in laboratory settings using experimentally induced pain, which may limit its ecological validity. To increase the clinical relevance of our study, we assessed the effects of VR distraction on the patients’ clinical pain and we used a commercially available tool. In this respect, it is interesting that we obtained large effect sizes using a nonimmersive VR system. Immersion refers to the objective characteristics of the technological system [50, 68], such as the occlusion from the outside world or the presence of a high definition panoramic view. Although research has shown that the level of immersion is related to the magnitude of the hypoalgesic effect of VR distraction [50, 69, 70], studies also indicate that mainly the interactive aspect of VR games may be responsible for this hypoalgesic effect [71–73]. As such, the latter might explain why the VR distraction in our study resulted in a significant reduction in pain, despite using a nonimmersive VR system.

To standardize the intervention, patients in the control group had to tilt their pelvis according to an auditory signal to ensure that they performed a similar number of repetitions compared to the VR group. As pelvic tilts can be painful for patients with CLBP, a temporal summation effect might occur if these movements are performed repetitively [74, 75]. It could be argued that the auditory signal might have distracted participants in the control group. However, as shown by the meta-analysis by van Ryckeghem et al. [2], non-VR distraction interventions do not have a hypoalgesic effect in patients with chronic pain. More specifically, Goubert et al. [15] reported that a tone detection task to distract patients with CLBP during an active task did not reduce the pain during this activity. Therefore, it is unlikely that the auditory signals in the control group resulted in a hypoalgesic effect.

Clinically, the aim of CLBP management is for patients to achieve their valued life goals [76]. Although different treatment strategies exist to obtain this aim, a common aspect is that they typically involve an active component, which may be embedded in a cognitive behavioral approach [77]. A major problem is that adherence to active therapies is low and the number of drop outs is high [56, 78], leading to suboptimal treatment results [56, 79, 80]. In this respect, patients with CLBP report that an important reason for this nonadherence is the experience of pain, or an increase of pain, during activities or exercises [56, 81]. Although participants in the current study only had to perform low load exercises for a few minutes, this already led to an increase in pain in the control group. Based on the planned contrasts (see Additional File 1), it is clear that this increase was mainly present in patients with higher levels of pain catastrophizing and pain-related fear. Since pain is an unpleasant experience, some patients are simply not willing to endure this temporary increase of pain [82]. Other patients report that engaging in painful activities can lead to increased (emotional) suffering that will interfere with their daily functioning [82]. As such, by reducing the pain intensity during exercises, VR games may have the potential to remove this barrier to engage in active therapies. From a clinical perspective it may also be important to integrate the VR-games into home exercises, as these are an essential aspect of the rehabilitation program for patients with CLBP. In a small feasibility study, we have previously shown that this is indeed possible for the games used in the current study [25]. Therefore, VR-games may provide an avenue for obtaining better long-term treatment results, potentially by increasing the adherence to exercises. Clearly, the latter needs further investigation.

Several limitations apply to this study. First, we assessed the pain immediately after the intervention, but it would be useful to also investigate the effects of VR distraction over a longer post-intervention period. This would allow to see whether a sustained hypoalgesic effect is present or, conversely, to detect a potential delayed rebound effect of VR distraction. Second, the majority of the participants in this study had a mild to moderate baseline pain intensity. This might have limited the potential to detect a moderating effect of this parameter on the VR distraction. Third, besides examining the moderating effects of pain-related fear and pain catastrophizing on the VR distraction, it would be of interest to use more direct measures of attention to pain, such as the Pain Vigilance and Awareness Questionnaire [83] or experimental methods [10, 11]. Fourth, because this study was not sufficiently powered to detect small effects for most moderation analyses, larger samples are required in future trials. Fifth, participants were familiar with pelvic tilts and did not perceive them as harmful. Given that the threat value and novelty of a stimulus can influence the attention to pain [12], future trials should investigate whether the hypoalgesic effects of VR distraction are also present during unfamiliar and threatening situations. Furthermore, it is possible that pain-related cognitions and emotions might have a moderating effect during these type of activities. Sixth, we did not control for situational anxiety. It is be possible that situational anxiety levels in the control group might have been elevated, because participants in the control group had to respond to beeps, which may have created a more unnatural situation compared to daily clinical practice. Furthermore, they might also have been more aware that they were being monitored by a researcher, since they were not being distracted. Finally, the participants and the researchers conducting the trial were not blinded during the intervention. Although we explicitly did not reveal any of our hypotheses to the patients (e.g. we expect VR to decrease the pain), it is possible that the lack of blinding might have influenced the results. However, it should also be noted that the effects of blinding on study results in the field of rehabilitation remain currently unclear [84, 85].

## Conclusions

In conclusion, this study provides evidence for the effectiveness of VR distraction to reduce the pain intensity during exercises for patients with CLBP. Pain-related fear, pain catastrophizing and baseline pain intensity did not moderate the hypoalgesic effects of VR distraction. Future trials should further investigate the role of the motivational aspects of distraction and specifically explore whether hypoalgesic effects are also present in patients with severe pain and during more threatening activities.

## Supplementary information

**Additional file 1.** Results of ANOVAs for moderation analyses. Results of ANOVAs for moderation analyses and the planned comparisons.

**Additional file 2.** : Results of the regression models for moderation analyses. Results of ANOVAs for moderation analyses.

**Additional file 3.** Power analyses of moderation effects. Power analyses of moderation effects.

**Additional file 4.** Consort checklist.

## Data Availability

The datasets used and/or analysed during the current study are available from the corresponding author on reasonable request.

## Abbreviations

CLBP: Chronic low back pain
LBP: Low back pain
IQR: Interquartile range
NPRS: Numeric pain rating scale
PCS: Pain Catastrophizing Scale
TSK: Tampa Scale for Kinesiophobia
VR: Virtual reality
