# Supplementary material for: Virtual reality distraction induces hypoalgesia in patients with chronic low back pain: a randomized controlled trial
Source: J Neuroeng Rehabil. 2020 Apr 22;17:55. doi: 10.1186/s12984-020-00688-0 (PMC7178732; doi:10.1186/s12984-020-00688-0)
Supplement: Supplementary file 1 — Additional file 1. Results of ANOVAs for moderation analyses. Results of ANOVAs for moderation analyses and the planned comparisons. [file 12984_2020_688_MOESM1_ESM.docx]

**Additional File 1 – Results of ANOVAs for moderation analyses**

***Influence on the difference between pain intensity at baseline and during the exercises***

Regarding pain-related fear, a main effect for group (F_(1, 80)_= 35.86, p< 0.0001, η_p_²= 0.31) and TSK (F_(1, 80)_= 11.80, p= 0.0009, η_p_²= 0.13) was present. There was no interaction effect (F_(1, 80)_ = 0.37, p= 0.54). Patients in the low TSK groups had larger improvements than those in the high TSK groups (Mean difference= 1.24, SE= 0.36). When assessing the influence of pain catastrophizing, there was a main effect for group (F_(1, 80)_= 36.47, p < 0.0001, η_p_²= 0.31) and pain catastrophizing (F_(1, 80)_ = 6.65, p= 0.01, η_p_² = 0.08) but no interaction effect (F_(1, 80)_= 0.01, p= 0.93). The improvements in the low PCS groups were larger than those in the high PCS groups (Mean difference= 0.95, SE= 0.37). For baseline pain intensity, there was only a significant main effect for group (F_(1, 80)_= 34.01, p < 0.0001, η_p_²= 0.29). No main effect for baseline pain intensity (F_(1, 80)_= 1.73, p= 0.19) and no interaction effect (F_(1, 80)_= 0.09, p= 0.75) were present. More detailed results can be found in Additional table 1.1

| **Additional table 1.1** Pain during exercises: Planned contrasts for low and high scores on baseline parameters | | | | | | |
| --- | --- | --- | --- | --- | --- | --- |
|  | Group | |  | Between group differences | | |
|  | Control | VR |  | F_(1, 80)_ | P | ES (η_p_²) |
| TSK |  |  |  |  |  |  |
| Low | -0.08 (0.34) | 2.29 (0.33) |  | 24.72 | < 0.0001 | 0.24 |
| High | -1.11 (0.38) | 0.83 (0.19) |  | 12.90 | 0.0006 | 0.14 |
| PCS |  |  |  |  |  |  |
| Low | -0.13 (0.35) | 2.13 (0.36) |  | 20.21 | < 0.0001 | 0.20 |
| High | -1.05 (0.39) | 1.15 (0.38) |  | 16.53 | 0.0001 | 0.17 |
| NPRS |  |  |  |  |  |  |
| Low | -0.86 (0.38) | 1.48 (0.38) |  | 18.88 | < 0.0001 | 0.19 |
| High | -0.24 (0.38) | 1.86 (0.38) |  | 15.23 | 0.0002 | 0.16 |
| Group means (SE) of the differences between pain intensity at baseline and during the exercises are presented. A negative value indicates an increase in pain intensity and a positive value indicates a decrease in pain intensity compared to baseline. NPRS: numeric pain rating scale, PCS= Pain Catastrophizing Scale, TSK= Tampa Scale for Kinesiophobia.  ***Influence on the difference between pain intensity at baseline and after the exercises***  For pain-related fear, there was a main effect for group (F_(1, 80)_= 17.34, p< 0.0001, η_p_²= 0.18) and for TSK (F_(1, 80)_= 19.04, p < 0.0001, η_p_²= 0.19), but no interaction effect (F_(1, 80)_= 0.13, p= 0.72). The low TSK groups had a larger improvement than the high TSK groups (Mean difference= 1.38, SE= 0.32). Regarding pain catastrophizing, a main effect for group (F_(1, 80)_= 16.45, p< 0.0001, η_p_²= 0.17) and PCS (F_(1, 80)_= 5.97, p< 0.02, η_p_²= 0.07) was present, but there was no interaction between these two factors (F_(1, 80)_= 0.31, p= 0.58). Participants in the low PCS groups experienced a larger improvement than participants in the high PCS groups (Mean difference= 0.82, SE= 0.34). Again, for baseline pain intensity there was only a main effect for group (F_(1, 80)_= 14.99, p= 0.0002, η_p_²= 0.16), but not for baseline pain intensity (F_(1, 80)_ = 3.86, p= 0.22), and no interaction effect was found (F_(1, 80)_< 0.01, p= 1.00). Details on the influence of the baseline parameters on the pain difference during exercises are presented in Additional table 1.2.   \| **Additional table 1.2** Pain after exercises: Planned contrasts for low and high scores on baseline parameters \| \| \| \| \| \| \| \| --- \| --- \| --- \| --- \| --- \| --- \| --- \| \|  \| Group \| \|  \| Between group differences \| \| \| \|  \| Control \| VR \|  \| F_(1, 80)_ \| P \| ES (η_p_²) \| \| TSK \|  \|  \|  \|  \|  \|  \| \| Low \| 0.17 (0.29) \| 1.38 (0.29) \|  \| 8.22 \| 0.005 \| 0.09 \| \| High \| -1.32 (0.33) \| 0.11 (0.34) \|  \| 9.14 \| 0.003 \| 0.10 \| \| PCS \|  \|  \|  \|  \|  \|  \| \| Low \| -0.04 (0.32) \| 1.14 (0.33) \|  \| 6.61 \| 0.01 \| 0.08 \| \| High \| -1.05 (0.35) \| 0.50 (0.34) \|  \| 9.92 \| 0.002 \| 0.11 \| \| NPRS \|  \|  \|  \|  \|  \|  \| \| Low \| -0.71 (0.35) \| 0.62 (0.35) \|  \| 7.45 \| 0.008 \| 0.09 \| \| High \| -0.29 (0.35) \| 1.05 (0.35) \|  \| 7.45 \| 0.008 \| 0.09 \| \| Group means (SE) of the differences between pain intensity at baseline and after the exercises are presented. A negative value indicates an increase in pain intensity and a positive value indicates a decrease in pain intensity compared to baseline. NPRS: numeric pain rating scale, PCS= Pain Catastrophizing Scale, TSK= Tampa Scale for Kinesiophobia. \| \| \| \| \| \| \|   ***Influence on the time spent thinking of pain***  Regarding pain-related fear, a main effect for group (F_(1, 80)_ = 37.06, p < 0.0001, η_p_²= 0.32) and TSK (F_(1, 80)_ = 5.67, p < 0.02, η_p_²= 0.07) was present, but there was no interaction effect (F_(1, 80)_ = 0.11, p= 0.74). Patients in the low TSK groups spent less time thinking of their pain compared to patients in the high TSK groups (Mean difference= 1.27, SE= 0.53). For pain catastrophizing, there was a main effect for group (F_(1, 80)_ = 42.46, p < 0.0001, η_p_²= 0.35) and PCS (F_(1, 80)_ = 14.09, p= 0.0003, η_p_²= 0.15), but no interaction effect was found (F_(1, 80)_ = 0.002, p= 0.97). The patients in the low PCS groups thought less of their pain than those in the high PCS groups (Mean difference= 1.91, SE= 0.51). Concerning baseline pain intensity, a main effect for group (F_(1, 80)_ = 37.83, p < 0.0001, η_p_²= 0.32) and baseline pain intensity (F_(1, 80)_ = 5.66, p < 0.02, η_p_²= 0.07) was present, but there was no interaction effect (F_(1, 80)_ = 0.10, p= 0.75). The low baseline pain groups spent less time thinking of their pain in comparison to the high groups (Mean difference= 1.27, SE= 0.53). More detailed results are shown in Additional table 1.3.   \| **Additional table 1.3** Time spent thinking of pain: Planned contrasts for low and high scores on baseline parameters \| \| \| \| \| \| \| \| --- \| --- \| --- \| --- \| --- \| --- \| --- \| \|  \| Group \| \|  \| Between group differences \| \| \| \|  \| Control \| VR \|  \| F_(1, 80)_ \| P \| ES (η_p_²) \| \| TSK \|  \|  \|  \|  \|  \|  \| \| Low \| 4.87 (0.51) \| 1.79 (0.50) \|  \| 18.84 \| < 0.0001 \| 0.19 \| \| High \| 6.32 (0.56) \| 2.89 (0.57) \|  \| 18.38 \| < 0.0001 \| 0.19 \| \| PCS \|  \|  \|  \|  \|  \|  \| \| Low \| 4.65 (0.48) \| 1.36 (0.49) \|  \| 22.59 \| < 0.0001 \| 0.22 \| \| High \| 6.58 (0.53) \| 3.25 (0.52) \|  \| 20.06 \| < 0.0001 \| 0.20 \| \| NPRS \|  \|  \|  \|  \|  \|  \| \| Low \| 4.81 (0.53) \| 1.71 (0.53) \|  \| 17.03 \| < 0.0001 \| 0.18 \| \| High \| 6.24 (0.53) \| 2.81 (0.53) \|  \| 20.89 \| < 0.0001 \| 0.21 \| \| Group means (SE) of the time spent thinking of pain during the exercises are presented. ES= Effect size, VR= virtual reality \| \| \| \| \| \| \| | | | | | | |
