# Supplementary material for: Virtual reality distraction induces hypoalgesia in patients with chronic low back pain: a randomized controlled trial
Source: J Neuroeng Rehabil. 2020 Apr 22;17:55. doi: 10.1186/s12984-020-00688-0 (PMC7178732; doi:10.1186/s12984-020-00688-0)
Supplement: Supplementary file 2 — Additional file 2. : Results of the regression models for moderation analyses. Results of ANOVAs for moderation analyses. [file 12984_2020_688_MOESM2_ESM.docx]

**Additional File 2 – Results of the regression models for moderation analyses**

| **Additional table 2.1** Regression models with continuous baseline measures for predicting the difference in pain during exercises | | | | | | | |
| --- | --- | --- | --- | --- | --- | --- | --- |
| Baseline measure | factors | Estimate | SE | t-ratio | p | R² adj | ΔR² adj |
| *Pain-related fear* | Group [C] | -1.14 | 0.18 | -6.53 | <0.0001 | 0.28 |  |
|  | TSK | -0.11 | 0.03 | -3.39 | 0.0002 | 0.38 | 0.10 |
|  | Group*TSK | 0.03 | 0.03 | 1.10 | 0.27 | 0.39 | 0.01 |
|  |  |  |  |  |  |  |  |
| *Catastrophizing* | Group [C] | -1.12 | 0.18 | -6.21 | <0.0001 | 0.28 |  |
|  | PCS | -0.06 | 0.02 | -3.13 | 0.002 | 0.36 | 0.08 |
|  | Group*PCS | 0.01 | 0.02 | 0.4 | 0.52 | 0.36 | 0.00 |
|  |  |  |  |  |  |  |  |
| *Baseline pain* | Group [C] | -1.11 | 0.19 | -5.92 | <0.0001 | 0.28 |  |
|  | NPRS | 0.19 | 0.13 | 2.26 | 0.14 | 0.29 | 0.01 |
|  | Group*NPRS | 0.16 | 0.13 | 1.66 | 0.20 | 0.30 | 0.01 |
| In the sequential multiple regression models, group was added in the first step, in the next step the baseline measure was added and in the final step the interaction between these two factors was added. The estimates (SE), t-ratio and p-values presented, are those for the full model. The difference in pain during the exercises (= dependent variable) was calculated by subtracting the pain intensity during the exercises from the baseline pain intensity. A positive value thus corresponds with an improvement in pain. Therefore, a negative estimate of a variable implies that this factor negatively influenced the improvement in pain intensity. Group [C]= Control group, NPRS= Numeric Pain Rating Scale, PCS= Pain Catastrophizing Scale, TSK= Tampa Scale for Kinesiophobia. | | | | | | | |

| **Additional table 2.2** Regression models with continuous baseline measures for predicting the pain difference after the exercises | | | | | | | |
| --- | --- | --- | --- | --- | --- | --- | --- |
| Baseline measure | factors | Estimate | SE | t-ratio | p | R² adj | ΔR² adj |
| *Pain-related fear* | Group [C] | -0.72 | 0.15 | -4.82 | <0.0001 | 0.14 |  |
|  | TSK | -0.14 | 0.02 | -5.54 | <0.0001 | 0.37 | 0.23 |
|  | Group*TSK | 0.02 | 0.02 | 0.99 | 0.32 | 0.37 | 0.00 |
|  |  |  |  |  |  |  |  |
| *Catastrophizing* | Group [C] | -0.68 | 0.16 | -4.12 | <0.0001 | 0.14 |  |
|  | PCS | -0.05 | 0.02 | -3.14 | 0.002 | 0.23 | 0.09 |
|  | Group*PCS | -0.002 | 0.02 | -0.13 | 0.90 | 0.22 | -0.01 |
|  |  |  |  |  |  |  |  |
| *Baseline pain* | Group [C] | -0.67 | 0.17 | -3.89 | 0.0002 | 0.14 |  |
|  | NPRS | 0.09 | 0.12 | 0.75 | 0.46 | 0.14 | 0.00 |
|  | Group*NPRS | 0.16 | 0.12 | 1.34 | 0.18 | 0.15 | 0.01 |
| In the sequential multiple regression models, group was added in the first step, in the next step the baseline measure was added and in the final step the interaction between these two factors was added. The estimates (SE), t-ratio and p-values presented, are those for the full model. The difference in pain after the exercises (= dependent variable) was calculated by subtracting the pain intensity after the exercises from the baseline pain intensity. A positive value thus corresponds with an improvement in pain. Therefore, a negative estimate of a variable implies that this factor negatively influenced the improvement in pain intensity. Group [C]= Control group, NPRS= Numeric Pain Rating Scale, PCS= Pain Catastrophizing Scale, TSK= Tampa Scale for Kinesiophobia. | | | | | | | |

| **Additional table 2.3** Regression models with continuous baseline measures for predicting the time spent thinking of pain | | | | | | | |
| --- | --- | --- | --- | --- | --- | --- | --- |
| Baseline measure | factors | Estimate | SE | t-ratio | p | R² adj | ΔR² adj |
| *Pain-related fear* | Group [C] | 1.68 | 0.26 | 6.39 | <0.0001 | 0.30 |  |
|  | TSK | 0.12 | 0.04 | 2.82 | 0.006 | 0.35 | 0.05 |
|  | Group*TSK | -0.03 | 0.04 | -0.75 | 0.46 | 0.35 | 0.00 |
|  |  |  |  |  |  |  |  |
| *Catastrophizing* | Group [C] | 1.65 | 0.26 | 6.47 | <0.0001 | 0.30 |  |
|  | PCS | 0.09 | 0.03 | 3.48 | 0.0008 | 0.39 | 0.09 |
|  | Group*PCS | -0.01 | 0.03 | -0.29 | 0.77 | 0.38 | -0.01 |
|  |  |  |  |  |  |  |  |
| *Baseline pain* | Group [C] | 1.61 | 0.26 | 6.24 | <0.0001 | 0.30 |  |
|  | NPRS | 0.56 | 0.18 | 3.15 | 0.002 | 0.37 | 0.07 |
|  | Group*NPRS | -0.04 | 0.18 | -0.20 | 0.84 | 0.36 | -0.01 |
| In the sequential multiple regression models, group was added in the first step, in the next step the baseline measure was added and in the final step the interaction between these two factors was added. The estimates (SE), t-ratio and p-values presented, are those for the full model. Group [C]= Control group, NPRS= Numeric Pain Rating Scale, PCS= Pain Catastrophizing Scale, TSK= Tampa Scale for Kinesiophobia. | | | | | | | |
