# Supplementary material for: Virtual reality distraction induces hypoalgesia in patients with chronic low back pain: a randomized controlled trial
Source: J Neuroeng Rehabil. 2020 Apr 22;17:55. doi: 10.1186/s12984-020-00688-0 (PMC7178732; doi:10.1186/s12984-020-00688-0)
Supplement: Supplementary file 3 — Additional file 3. Power analyses of moderation effects. Power analyses of moderation effects. [file 12984_2020_688_MOESM3_ESM.docx]

**Additional File 3 – Power analyses for the moderation effects**

| Power analyses of moderation effects | | | | | |
| --- | --- | --- | --- | --- | --- |
| Measure | Outcome | Current ES (η_p_²) | Power for current ES |  | Detectable ES (η_p_²) |
| TSK | Pain difference during | 0.00463 | 0.09 |  | 0.028 |
|  | Pain difference post | 0.0016 | 0.06 |  | 0.016 |
|  | Time thinking | 0.0013 | 0.06 |  | 0.015 |
|  |  |  |  |  |  |
| PCS | Pain difference during | 0.00009 | 0.05 |  | 0.004 |
|  | Pain difference post | 0.0038 | 0.08 |  | 0.025 |
|  | Time thinking | 0.00002 | 0.05 |  | 0.001 |
|  |  |  |  |  |  |
| Baseline pain | Pain difference during | 0.00123 | 0.06 |  | 0.014 |
|  | Pain difference post | - | <0.05 |  | - |
|  | Time thinking | 0.00123 | 0.06 |  | 0.014 |
| The current ES is the ES obtained for the moderation effects in the current study, based on the ANOVAs. The power for detecting these effect sizes is provided (Power for current ES). The detectable ES is the effect size that would be detectable, based on the current sample size and results of this study, with α= 0.05 and power= 0.95. Effect sizes and power could not be calculated for the moderation effects of baseline pain on the pain-difference during exercises (Results ANOVA= F_(1, 80)_< 0.01, p= 1.00).  ES= Effect size (η_p_²), PCS= Pain Catastrophizing Scale, TSK= Tampa scale for Kinesiophobia | | | | | |
